# Dissolution

**Published:** 1881-10

**Authors:** 


### DISSOLUTION

The partnership existing for the past six years between J. & C. R. Taft, in the practice of Dentistry in Cincinnati, is by mutual consent dissolved. Said dissolution takes effect October 15, 1881.

